# Overexpression of CTLA-4 and fibronectin, and lower expression of CD137 (4-1BB) is associated with brain metastasis of primary skin melanomas. An analysis of local immune response by digital spatial profiling

**DOI:** 10.3389/fimmu.2025.1578986

**Published:** 2025-06-20

**Authors:** Shiva Najjary, Nur al Amery, Mengqi Huang, Disha Vadgama, Alexander C. J. van Akkooi, H. Jacobus Gilhuis, Zineb Belcaid, Dana Mustafa, Johan M. Kros

**Affiliations:** ^1^ Department of Pathology, Erasmus Medical Center, Rotterdam, Netherlands; ^2^ Melanoma Institute Australia, Sydney, NSW, Australia; ^3^ Faculty of Medicine and Health, University of Sydney, Sydney, NSW, Australia; ^4^ Department of Melanoma and Surgical Oncology, Institute of Academic Surgery, Royal Prince Alfred Hospital, Sydney, NSW, Australia; ^5^ Department of Neurology, Reinier de Graaf Ziekenhuis, Delft, Netherlands

**Keywords:** melanoma, metastasis, CTLA-4, fibronectin, spatial biology, 4-1BB, immune response

## Abstract

**Background:**

To discover immune response-associated gene expressions related to brain metastases of skin melanomas, we analyzed a group of fifteen primary skin melanomas consisting of five tumors from patients who did not develop systemic metastases (NM); ten from patients with systemic metastasis of which five with (BM), and five without brain involvement (OOM). Regions of interest (ROIs) harboring tumor cells only; immune cells only; or immune cell infiltration in tumor cell regions, were separately analyzed.

**Methods:**

we profiled the tumor immune microenvironment (TIME) of primary skin melanomas by using a panel of 77 immune response-related oligo-nucleotide targets in the GeoMx Digital Spatial profiler (Nanostring Technologies).

**Results:**

Melanomas from patients who developed brain metastases contained significantly higher levels of (CD45+) immune cells and overexpressed fibronectin and CTLA-4 in all ROIs compared to the melanomas that had metastasized to other sites than the brain (p<0.05). Distant metastases were accompanied by lower expression of CD137 (4-1BB). Downregulation of VISTA, IDO1 and ICOS was associated with melanomas that gave rise to distant metastases including brain.

**Conclusion:**

Lower expression of CD137 (4-1BB) is linked with the formation of distant metastases and the expressions of fibronectin and CTLA-4 herald the formation of brain metastasis in particular.

## Introduction

The high mortality of melanoma patients is foremost caused by metastasis to distant organs. No less than 40-60% of patients will develop brain metastasis (BM) in the course of the disease (1) and with this complication of the disease patients have the worst survival rates (2, 3). Despite the improved outcomes of newly developed immunotherapies (4, 5), recurrent disease is still a major problem. Since the development of BM accounts for about 50% of all melanoma deaths (6) it is of vital importance to identify the key players in the formation of cerebral seeding to be able to develop targets for BM preventing strategies. Over recent years particular immune cells in the tumor immune microenvironment (TIME) were traced that promote tumor progression and organ-specific metastasis (7–10). Tumor cells exploit immune mechanisms in the primary tumor to escape elimination in the blood stream and to home at distant sites. Upon their passing through the blood-brain barrier (BBB) the tumor cells must deal with the local brain immune microenvironment that differs from that of the primary tumors (10–12). The TIME of the primary tumors contains distinct immune cells including neutrophils, mast cells, T and B lymphocytes, natural killer cells, and antigen-presenting cells (APCs). While adaptive immune cells such as NK cells and CD8+ T lymphocytes may inhibit tumor growth by promoting anti-tumor immunity (13), several studies have demonstrated that innate immune cells, such as macrophages, mast cells, and neutrophils are capable of forming a chronic inflammatory environment that promotes epithelial-mesenchymal transition (EMT) and neovascularization, which are essential processes for tumor progression (7, 14). Increased T cell infiltration was found to be beneficial to patient survival in numerous tumor types including melanoma (15, 16). It also appears that particular immune cells may display contradictory actions in the dissemination of primary tumors. For example, the ability of T lymphocytes to enhance the migratory capacity of breast cancer cells was demonstrated using both an *in vitro* BBB model as well as an *in vivo* mouse model (9). In order to elucidate the underlying mechanisms involved in the development of melanoma brain metastasis (MBM) and the role and effects of particular immune cells on tumor cell behavior, exploration of the TIME immune landscape is crucial. Importantly, parameters in the TIME that would be predictive of the formation of metastasis, particularly brain metastasis, would be important for therapeutic strategies for patients suffering from melanomas.

In this study we characterize the immune response in regions in and around primary skin melanomas of patients with and without distant metastases including brain metastases. We compared the TIME of primary melanomas obtained from patients who did not have metastases (NM); patients who had developed BM exclusively or in addition to metastases to other organs, and patients who developed metastases in organs other than the brain (OOM). To that aim, we utilized the GeoMx Digital Spatial Profiler (DSP) for multiplex protein detection in formalin-fixed paraffin-embedded (FFPE) tissue sections, providing quantitative and spatial information. The expression of a large set of proteins within regions of interest (ROIs), selected by standard fluorescent IHC on single tissue slides were quantified (17, 18).

## Materials and methods

### Patient cohorts and tumor characteristics

This study was approved by the Medical Ethics Committee of the Erasmus Medical Center, Rotterdam, the Netherlands, and was carried out in adherence to the Code of Conduct of the Federation of Medical Scientific Societies in the Netherlands (MEC 02·953 & MEC-2020–0732). To study the TIME associated with the formation of BM, treatment naïve primary malignant melanoma samples were selected from a patient cohort with completed follow-up. In this cohort, a total of 15 primary human melanoma FFPE tissues were selected from five patients who had developed distant metastasis without brain involvement (OOM); five patients who had developed brain metastases (BM) and five patients who had not developed metastases (NM). All patients had been admitted between 2000 and 2006 and none of them had received immune therapy, chemotherapy or therapy directed to BRAK/MEK mutations. The clinical features including patient characteristics, follow-up and tumor characteristics are listed in **Table 1**. The mean ages and age ranges of patients in the three metastatic groups were comparable. There was no gender skew. Melanoma histologic subtypes (superficial spreading, nodular and spindle cell) were all present in the BM group, while the spindle cell subtype was absent from the OOM group and the nodular subtype was absent from the group without metastases. The mean Breslow thickness of the 15 selected cutaneous melanomas for the NM, OOM and BM groups were 5.06, 3.37 and 4.18, respectively. The time period between the diagnosis of the primary tumor and the appearance of the first metastasis ranged from 18 to 71 months for the OOM group and from 2 to 99 months for the BM group. The sites of the primary tumors were divided into head/neck region, trunk and limbs. There was only one patient with head/neck location in the NM group. For the BM group 4/5 melanomas were located in the limbs. Most frequent metastatic sites other than brain included lymph nodes, lung, bone and liver. All tumors carried a BRAF mutation.

**Table 1:** Clinico-pathological characteristics of the cohort.

Characteristics	All patients (N=15)	NM (N=5)	OOM (N=5)	BM (N=5)
*Patient Characteristics*				
Age (years), mean (range)	49.6 (25-69)	51.0 (30-61)	49.2 (25-60)	48.6 (34-69)
Sex				
Male (%)	7 (46.7)	2 (40)	3 (60)	2 (40)
Female (%)	8 (53.3)	3 (60)	2 (40)	3 (60)
*Tumor characteristics*				
Breslow, mm	4.82	5.06	3.37	4.18
Ulceration				
Absent (%)	8 (53.3)	3 (60)	3 (60)	1 (20)
Present (%)	7 (46.7)	2 (40)	2 (40)	4 (80)
Unknown (%)	–	–	–	–
Histology				
SSM* (%)	8 (53.3)	4 (80)	2 (40)	2 (40)
NM** (%)	5 (33.3)		3 (60)	2 (40)
SCM*** (%)	2 (13.3)	1 (20)		1 (20)
Tumor location				
Limb (%)	7 (46.7)	1 (20)	2 (40)	4 (80)
Trunk (%)	7 (46.7)	3 (60)	3 (60)	1 (20)
Head/Neck (%)	1 (6.7)	1 (20)	–	–
*Outcome*				
Time to distant metastasis, months, mean (range)	N/A	N/A	41.2 (18-71)	40.8 (2-99)
Sites of distant metastasis				
Multiple organs(%)			5 (100)	2 (40)
Lymph node (%)			5 (100)	5 (100)
Brain (%)			–	5 (100)
Lung (%)			2 (40)	1 (20)
Bone (%)			1 (20)	1 (20)
Liver (%)			3 (60)	1 (20)
Other (%)			5 (100)	1 (20)

(*) SSM, Superficial Spreading Melanoma; (**) NM, Nodular Melanoma; (***) SCM, Spindle cell melanoma.

### Immunohistochemical stainings

Sections of 4 μm thickness were processed from the FFPE blocks and stained with routine H&E while immunohistochemistry to S100 and CD45 was carried out on adjacent sections according to the manufacturer’s protocols. S100 immunohistochemistry was used for the identification of the melanoma cells while the immune cells were identified by CD45+ immunostaining. The CD45 positive cells in the intra/extra-tumoral regions were quantified and compared between primary melanoma tissues based on their metastatic status by VisioPharm image analysis (Visiopharm, Hørsholm, Denmark). Immune cells and targets identified in DSP were quantified using VisioPharm software version 2018.9.

### Regions of interest

The respective ROIs were defined as tumor-rich without immune cells (S100+); immune cell-rich without tumor cells (CD45+) and tumor infiltrated by immune cells (S100+/CD45+). The CD45+ immune cells in and outside of the S100+ tumor areas were quantified. Per slide 3 ROIs were selected. Thus, for each metastatic group (NM, OOM and BM) 15 ROIs were selected.

### GeoMx Nanostring Digital Spatial profiling: protein expression

The protein expression was profiled using the GeoMx DSP. In brief, one FFPE tissue section of 5µ from FFPE blocks was stained with immunofluorescent antibodies: DNA stain (Syto13), melanoma cells (S100), and leukocytes (CD45). Simultaneously, slides were incubated with a cocktail of 77 UV-photocleavable oligonucleotide-labeled antibodies, which included immune-related targets, housekeeping proteins, and negative controls (**Table 2**). The protein expression profiles were processed prior to the analysis. The expression of twelve out of the 77 proteins from the analysis was detected in less than 50% of ROIs.

**Table 2:** UV-Photocleavable oligonucleotide-labeled antibody cocktail modules.

Immune Cell Profiling Core	IO Drug Target Module	Immune Activation Status Module	Immune Cell Typing Module	Pan-Tumor Module	PI3K-AKT Module	Cell Death Module
PD-1	4-1BB	CD127	CD45RO	MART1	Phospho-AKT1 (S473)	BAD
CD68	LAG3	CD25	FOXP3	NY-ESO-1		BCL6
HLA-DR	OX40L	CD80	CD34	S100	Phospho-GSK3B (S9)	BCLXL
Ki-67	Tim-3	ICOS	CD66b	Bcl-2		CD95/Fas
Beta-2- microglobulin	VISTA	PD-L2	FAP-alpha	EpCAM	Phospho-GSK3A (S21)/Phospho-GSK3B (S9)	GZMA
	ARG1	CD40	CD14	Her2		Cleaved Caspase 9
CD11c	B7-H3	CD44	CD163	PTEN		p53
CD20	IDO1	CD27		ER-alpha	INPP4B	PARP
CD3	STING			PR	PLCG1	BIM
CD4	GITR				Phospho-PRAS40 (T246)	
CD45						
CD56					Phospho-Tuberin (T1462)	
CD8					Pan-AKT	
CTLA-4					MET	
GZMB					Phospho-AKT (T308)	
PD-L1						
PanCk						
SMA						
Fibronectin						
Rb IgG						
Ms IgG1						
Ms IgG2a						
Histone H3						
S6						
GAPDH						

### Artificial intelligence-based image analysis and oligo detection

The Multiplex Phenotyping Module in the Visiopharm software (Visiopharm, Hørsholm, Denmark) was employed to analyze the whole-slide immunofluorescent DSP images. In order to prevent interference and ensure accuracy, image processing excluded areas with erythrocytes and strong fluorescent intensities. The software was trained to recognize S100+ (tumor) regions and CD45+ immune cell infiltrations. Additionally, the software was utilized for quantifying CD20+, CD27+, CD68+, HLA-DR+ and CTLA-4+ cells in immunofluorescent DSP validation images, following similar procedures. The counts of CD45, CD20, CD27, CD68, Human Leukocyte Antigen (HLA)-DR, and Cytotoxic-T-Lymphocyte Associated Protein 4 (CTLA-4) were normalized by total cell numbers within the corresponding intra/extra-tumoral regions to obtain relative cell counts. For the validation experiments, FFPE slides were only incubated with CD20, CD27, CD68, DR (HLA-DR) or (CTLA-4) visualization antibodies. Lastly, the photocleaved oligos released from the ROIs were hybridized for 17 hours at 65°C and scanned using the nCounter^®^ system (Nanostring Technologies, Seattle, WA, United States). The data were analyzed using DSP software.

### Statistical analysis

Protein expression differences between groups were evaluated using GeoMx DSP Analysis software version 2.4.0.147. The data were normalized based on the housekeeping proteins S6 and Histone H3 and corrected for the background determined by subtracting the expression of the negative control Ms.IgG2a in each ROI. The significant differences in the protein expression (adj. p ≤ 0.05) between the groups were calculated by linear mixed modeling (LMM). The relative positive cell counts were calculated by dividing the number of CD45, CD20, CD27, CD68, HLA-DR and CTLA-4 by the total number of cells in the corresponding tissue regions. All statistical analyses were performed using R statistical software (version 4.0.1). Two-sided P-values were considered, and statistical significance was set at P-value ≤ 0.05. The web-based tool Morpheus by Broad Institute (RRID: SCR_017386) was used for the visualization of data as heatmaps.

## Results

### Cell type-specific differences

**Figure 1:**
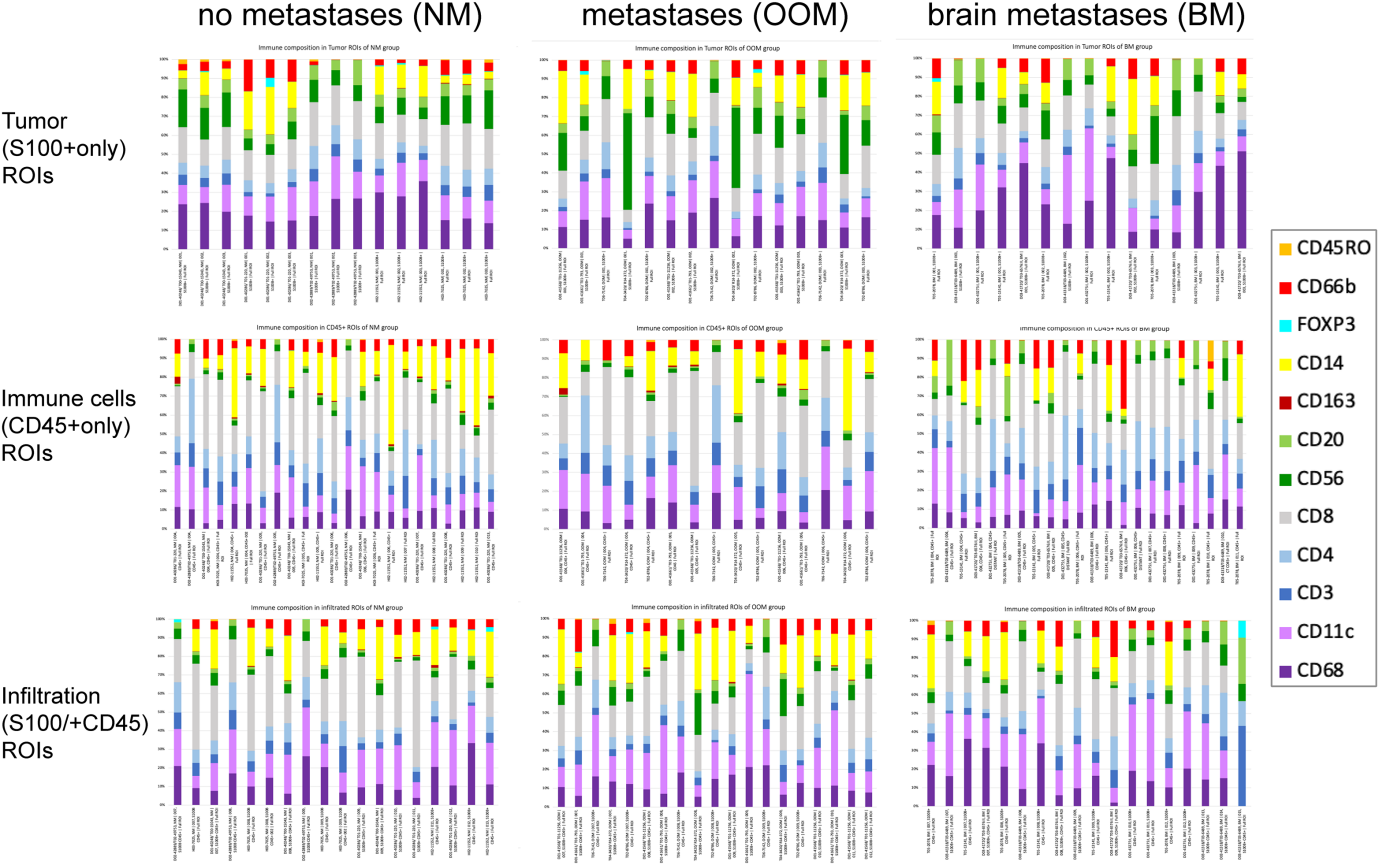
Immune cell composition of the ROIs for the melanomas with the different metastatic status. Each bar shows the proportion of immune cells in each ROI associated with the distinct metastatic categories of melanoma (NM, OOM, BM). There is some variation in the number of ROIs analyzed per category. For further comments see Results. .

The cellular compositions of the immune infiltrates in the various ROIs are shown in **Figure 1**. Melanomas from the NM and BM groups contained significantly higher levels of (CD45+) immune cells in all ROIs compared to the melanomas from the OOM group (p<0.05). In the tumor (S100+) ROIs of the BM group, less CD56 expression and more CD68 and CD11c expression was present than in the OOM group. In the immune cell (CD45+) ROIs of the BM group more CD20+ and CD56+ cells were found while the CD45+ ROIs of the NM and OOM groups contained slightly more CD14+ cells. The immune cell-infiltrated tumor areas (S100+/CD45+ ROIs) in the NM and OOM groups contained more CD14+ and more CD66b+ cells compared to the BM group.

### Analysis of ROIs

Most expressional differences between the melanoma metastatic groups were found between the tumors with brain metastases (BM) and tumors without metastases (NM) (**Table 3**). Least differences were found between melanomas without metastases and the tumors with metastases at sites other than brain (**Table 3**).

**Table 3:** Genes differentially expressed in the respective ROIs between the melanoma groups with different metastatic states.

	BM vs. NM			BM vs. OOM			OOM vs. NM		
ROIs	S100	CD45	S100/CD45	S100	CD45	S100/CD45	S100	CD45	S100/CD45
RNAs									
fibronectin									
CTLA-4									
4-1BB									
IDOI									
VISTA									
ICOS									
OX40L									
CD163									
CD27									
GITR									
PR-α									
EPCAM									
ER									
panCK									
MART1									

BM, melanomas with brain metastases; NM, melanomas without distant metastases; OOM, melanomas with distant metastases without brain involvement. ROI, region of interest. S100 = marker for melanoma cells; CD45 = marker for immune cells; S100/CD45 = areas of tumor with infiltrating immune cells. Green box: upregulation; red box: downregulation for the various comparisons.

### Overexpression of CTLA-4, CD27, fibronectin and lower expression of 4-1BB and OX40L in S100+ ROIs is associated with brain metastasis

**Figure 2:**
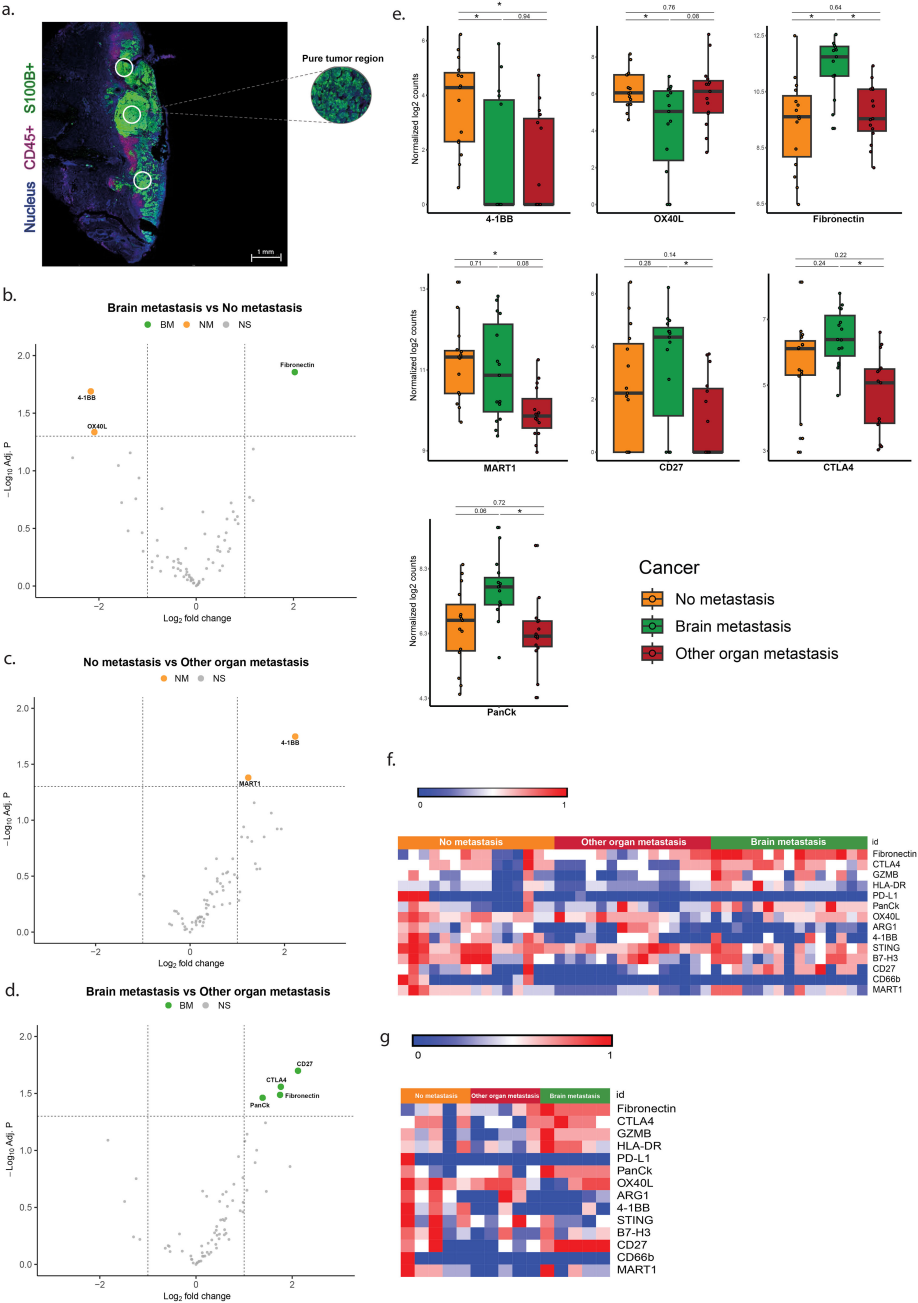
Differentially expressed genes in the ROIs of S100+ melanoma without infiltration by immune cells. **(a)** Immunofluorescent image of the S100+ pure tumor cell regions. **(b–d)** Vulcano plots of the analysis of expressional differences between the metastatic categories of melanomas. **(e)** Bar diagrams of the mean/s.e.m. of the expressional comparisons between the melanoma metastatic categories. **(f)** Heat map of normalized expression data **(g)** Heatmap of median normalized expression data. For comments see Results * = p<0.05.

Analysis of tumor (S100+) ROIs revealed significant overexpression of fibronectin and lower expression of 4-1BB and OX40L between the group with brain metastases (BM) versus the group without metastases (NM) (**Table 3**, **Figures 2b, e, f**). In addition, melanomas with brain metastases (BM) showed higher expression levels of fibronectin, CTLA-4, CD27, and PanCK compared to tumors with metastases in other organs (OOM) (**Table 3**, **Figures 2d–f**). The tumors without metastases (NM) showed higher expression levels of 4-1BB and MART1 compared to those with distant metastases without brain involvement (OOM) (**Table 3**, **Figures 2c, e, f**).

### Overexpression of CTLA-4 and lower expression of 4-1BB, IDO1, CD163, GITR, PR-α, EPCAM and ER in CD45+ ROIs is associated with brain metastasis

**Figure 3:**
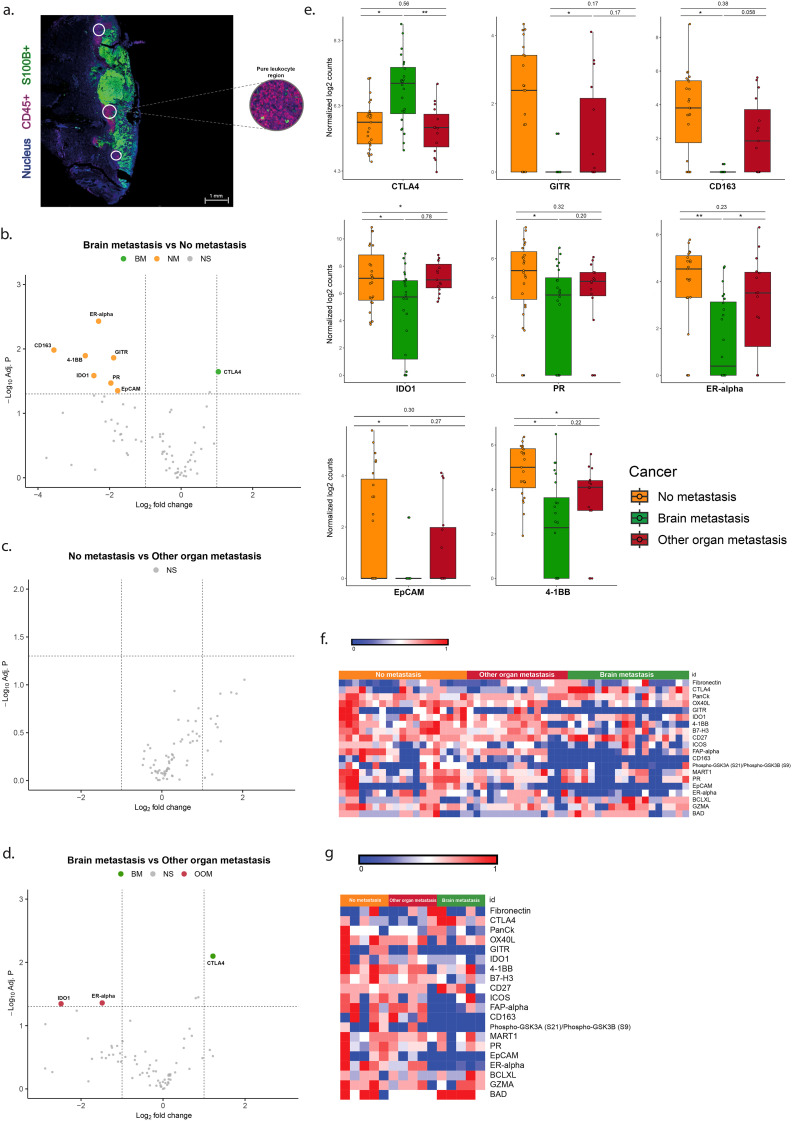
Differentially expressed genes in the ROIs of CD45+ immune cells without tumor cells. **(a)** Immunofluorescent image of the S100+ pure tumor cell regions. **(b–d)** Vulcano plots of the analysis of expressional differences between the metastatic categories of melanomas. **(e)** Bar diagrams of the mean/s.e.m. of the expressional comparisons between the melanoma metastatic categories. **(f)** Heat map of normalized expression data **(g)**. Heatmap of median normalized expression data. For comments see Results. * = p<0.05, ** = p<0.01.

Analysis of the pure immune cell (CD45+) ROIs without (S100+) tumor cells is shown in **Figure 3**. The melanomas with brain metastases (BM) showed higher expression of CTLA-4, and lower expression of 4-1BB, CD163, GITR, IDO1, PR, EPCAM and ER-alpha than the melanomas without metastases (**Table 3**, **Figures 3b, e, f**). In the BM group more expression of CTL4, and less expression of IDO1 and ER-alpha was found compared to the OOM group (**Figures 3d–g**). No expressional differences were found between the melanomas without metastases (NM) and those with metastases in organs (**Figures 3c, f**).

### Overexpression of CTLA-4 and ICOS, and lower expression of IDO1 and VISTA in CD45+/S100+ ROIs is associated with brain metastasis

**Figure 4:**
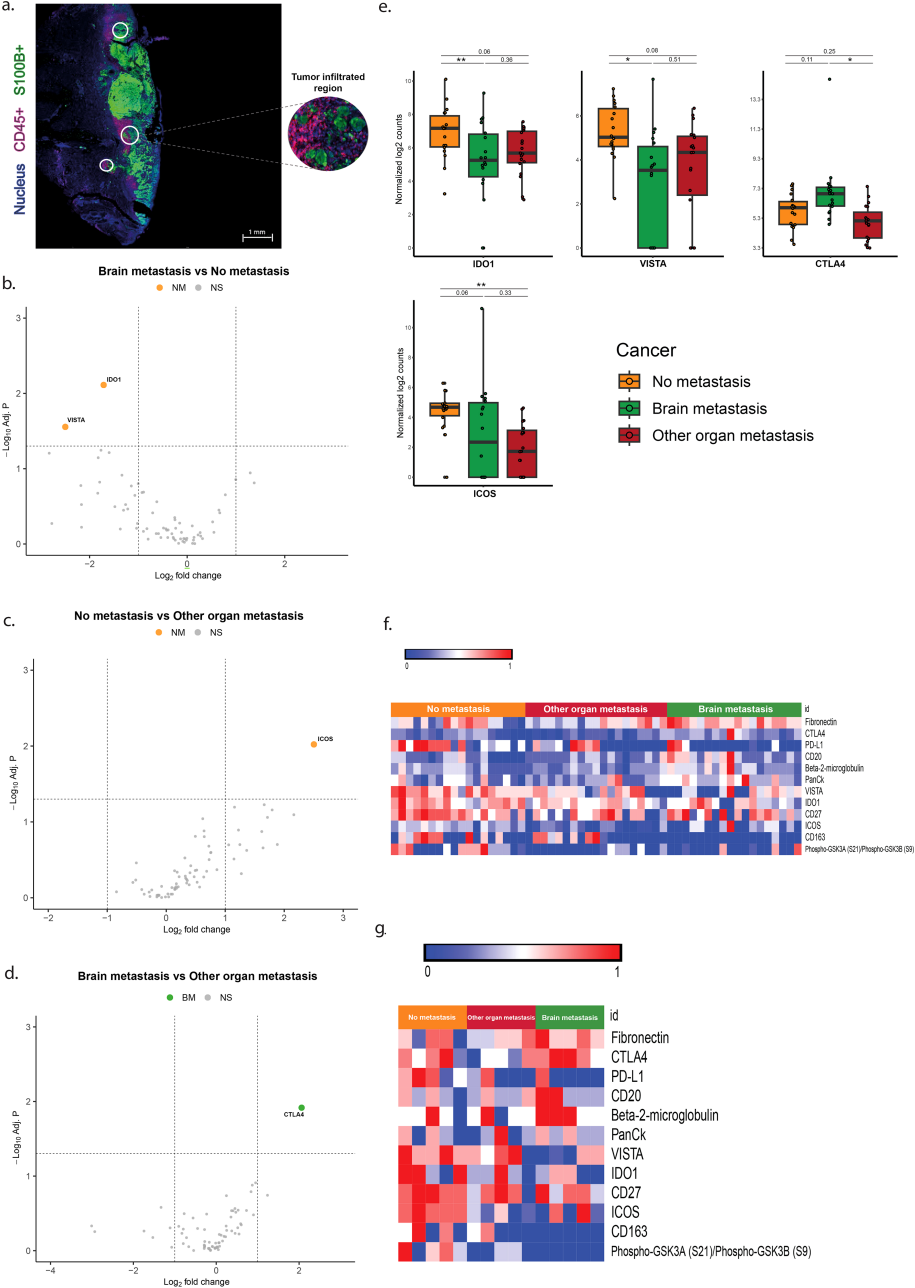
Differentially expressed genes in the ROIs of S100+ tumor areas infiltrated by CD45+ immune cells. **(a)** Immunofluorescent image of the S100+ pure tumor cell regions. **(b–d)** Vulcano plots of the analysis of expressional differences between the metastatic categories of melanomas. **(e)** Bar diagrams of the mean/s.e.m. of the expressional comparisons between the melanoma metastatic categories. **(f)** Heat map of normalized expression data **(g)**. Heatmap of median normalized expression data. For comments see Results. * = p<0.05, ** = p<0.01.

In the areas with tumor fields infiltrated by immune cells (CD45+/S100+ ROIs) melanomas with brain metastases (BM) showed lower expression of VISTA and IDO1 and no overexpression of genes when compared to tumors without metastases (NM) (**Table 3**, **Figures 4b, e, f**). In these ROIs tumors of the BM group overexpressed CTLA-4 compared to melanomas from the OOM group (**Table 3**, **Figures 4d–f**). Melanomas without metastases (NM) showed more expression of ICOS compared to tumors from the OOM group (**Table 3**, **Figures 4c, e, f**).

## Discussion

So far, controversial data on the impact of inflammatory infiltrates in the primary lesions on the metastatic status and prognosis of melanoma patients exists (19–21). Data on the composition of the immune infiltrates and prominent molecules acting in the immune response in regions in- and outside of the tumor fields are scarce (22, 23). From the literature the relation between the characteristics of the immune response with metastatic spread, specifically to brain, remains obscure because in the various studies different primary tumor localizations were included and the location of the immune cells in relation to the tumor cells is rarely specified. In the present study we specifically compared the immune infiltrations in primary skin melanomas, specified the sites of the immune cells and related their expressional repertoires to the development of distant metastases (OOM), particularly brain metastases (BM). When characterizing the TIME for immune response-related molecules by protein GeoMx DSP and validation of the findings by immunofluorescence imaging, we found striking differences in the compositions of the primary melanoma TIMEs between the patients with different metastatic outcomes.

In melanomas with brain metastases the expression of cytotoxic T-lymphocyte-associated protein 4 (CTLA-4; CD152) detected in the immune cell (CD45+) ROIs was higher than in tumors without metastases or metastases at other sites. CTLA-4 blocks CD28-mediated T cell activation, thereby functioning as an immune checkpoint inhibitor of T cell function. This immune checkpoint inhibitor is regarded as a reliable target in the treatment of cancer including advanced melanoma (24). CTLA-4 is targeted by the use of anti-CTLA-4 antibodies blocking the inhibitory signals from the effector T cells, allowing co-stimulatory signaling and activating T cells. In 2011, the use of anti-CTLA-4 was first approved drug by the U.S. food and drug administration (FDA) for the treatment of advanced melanoma patients, based on a significant improvement in overall survival (25). More recent studies with immunotherapies have shown efficacy of these drugs, also within the brain (4, 5). In the light of our previous observation that activated T cells play a role in the development of cerebral metastases in breast cancer patients, specific data on the development of brain metastases in the melanoma cohorts are of interest (9).

In the present analysis high expression of fibronectin in primary melanomas associated with BM was also observed. Specifically, fibronectin overexpression was found in the S100+ tumor cell regions. Fibronectin is one of the most important components within the extracellular matrix (ECM) as it is responsible for the communication between the intra- and extracellular environment (26). High expression of fibronectin is tightly correlated with invasive and metastatic behavior of melanoma cells (27–30). Besides melanoma, fibronectin has also been suggested to play a pro-metastatic role in other cancer types including cancers from colon (31), esophagus (32), lung (33) and breast (34). The pro-metastatic role of fibronectin upregulation in these tumors is the result of ECM remodeling allowing cancer cell migration and invasion (35). The present observation suggests that high expression of fibronectin not only leads to the remodeling of the melanoma ECM which in turn promotes the dissemination of tumor cells from the primary tumor, but also facilitates their entry in the brain. Overexpression of fibronectin at the site of the primary tumor may well facilitate tumor cells entering the blood stream, but the relation of fibronectin with specific seeding to brain needs further exploration (36).

Our results showed distinct downregulation of 4-1BB (CD137/TNF receptor superfamily SF9) in the tumor areas infiltrated by immune cells (S100+/CD45+ ROIs) of melanomas with distant metastasis including brain. 4-1BB is a receptor that activates various signaling pathways involved in the cellular immune response, providing metabolic support to T cells by enhancing the mitochondrial oxidative phosphorylation. Various immune cells including (skin) fibroblasts express 4-1BB. In addition, 4-1BB expression has been found in colonic cancer cells where it is also associated with the rise of distant metastases (37). With lower activity of 4-1BB the immune defense is hampered, allowing tumor cells to spread. Although the exact sequels of stimulating 4-1BB action is not completely understood, boosting 4-1BB action by agonistic antibodies is considered as a promising strategy to anti-cancer immunotherapy (38) and should be considered in the treatment of melanoma as well. Immune activating molecules other than 4-1BB like OX40 and GITR were also found downregulated and therefore, the effects of agonistic antibodies against these molecules need further evaluation (36, 39, 40). Downregulation of the immune checkpoint molecules IDO1 and VISTA was specifically observed in the ROIs with CD45+ immune cells, while not in the S100+ tumor fields, which is in line with the notion that VISTA is expressed by TIL subsets, and interaction between tumor cells and immune cells is necessary for its expression. Lower expression of VISTA was specifically noticed in tumors with brain metastases (**Table 3**). Therapies using monoclonal antibodies targeting VISTA result in slowing down tumor progression. It remains unclear why tumors from patients with BM are associated with lower VISTA expression. The current data warrant further analysis in prospective settings to evaluate the role of IDO1 and VISTA in metastasis particularly to the brain.

We found overexpression of CD27 in the (S100+) tumor fields of melanomas specifically seeding to brain (BM) as compared to tumors metastasizing to other organs (OOM). CD27 is a membrane glycoprotein that functions as a T cell co-stimulatory molecule in T cells. Upon antigen presentation, T cell expression of CD27 increases the early stages of the adaptive immune response. Abundant infiltration of activated T cells in melanoma has been associated with more favorable prognosis (41, 42). While activation of CD27 in a murine melanoma model reduced the development of distant metastasis (43), in human ER-negative breast cancers activated T cells were shown to promote the rise of brain metastases (9). Moreover, the present findings are also suggestive of a correlation between abundant active T cell infiltration and the development of BM.

In primary melanomas associated with development of cerebral metastasis lower expression levels of CD163 were observed. Brain metastasis was associated with peritumoral, not intratumoral, presence of CD68+ TAMs (10.1097/CMR.0b013e3283252feb PLEASE HELP; REF FOUND BUT IS NOT IN PUB MED). Macrophages residing in tumors show expressional profiles anywhere between polarities known as the M1 and M2 extremes. The M1 profile exerts pro-inflammatory effects thereby suppressing cancer progression. In contrast, the M2 profile is an anti-inflammatory macrophage with tumor-promoting capabilities involving immune-suppression, angiogenesis and neovascularization (44–46). Several studies on solid tumors including melanoma, have demonstrated that the abundant presence of tumor-associated macrophages (TAMs), in particular M2 macrophages, is associated with increased tumor progression and worse overall prognosis (46–48). To understand the role of the diminished expression of CD163 in macrophages in the dissemination of melanoma cells to the brain, specific explorations to determine the role of macrophage- and monocyte subtypes in the development of brain metastases are required.

There are clear limitations to the present study. The set-up is retrospective and should be validated in a prospective setting. At this point, the current results provide a frame of reference for larger spatial proteomic analyses to elucidate the role of the TIME in the development of BM. In addition, functional assays testing the effects of particular immune cell populations and testing the impact of blocking or overexpression of particular inflammatory molecules should validate the results from functional perspective. Such supporting studies are essential for the identification of new therapeutic opportunities to prevent formation of cerebral metastasis and improve care of patients with melanoma.

## Data Availability

The nanostring data / results deposited at the directories of the Erasmus MC, Rotterdam, The Netherlands, and are available on request from the corresponding author(s).
